# Impact of ADHD Subtypes, Cognitive Disengagement Syndrome and Anxiety on Memory Functions and Visuospatial Skills in Children with ADHD

**DOI:** 10.1192/j.eurpsy.2025.448

**Published:** 2025-08-26

**Authors:** I. Adak, E. Özdeniz Varan, Ö. Ekinci, Z. Durmuş, N. Kılıç, A. Alpman, O. B. Karakuş, I. Süzer Gamlı

**Affiliations:** 1 Child and Adolescent Psychiatry, Erenköy Mental and Nervous Diseases Training and Research Hospital; 2 Clinical Psychology, University of Health Sciences; 3 Child and Adolescent Psychiatry, Trabzon Provincial Health Directorate Kanuni Training and Research Hospital, İstanbul, Türkiye

## Abstract

**Introduction:**

Children with Attention Deficit Hyperactivity Disorder (ADHD) may experience impairments in memory functions and visuospatial skills. Despite this, research exploring the relationship between ADHD subtypes, psychiatric comorbidities, and these neurocognitive functions is limited. This study focused on cognitive disengagement syndrome (CDS) and anxiety as comorbid conditions, both of which independently impact neurocognitive functions. However, the combined effects of these conditions with ADHD remain poorly understood. Moreover, investigating how CDS influences neurocognitive domains is essential for a comprehensive understanding of the syndrome’s cognitive aspects. Therefore, identifying the factors that impact memory functions and visuospatial skills in children with ADHD is expected to facilitate the recognition of cases at risk.

**Objectives:**

This study aimed to investigate the relationship between ADHD subtypes, psychiatric comorbidities, and memory functions and visual spatial skills in children with ADHD.

**Methods:**

The study recruited 120 children with ADHD aged 6-12 years. ADHD subtypes, anxiety, and CDS symptoms were assessed using standardized scales and DSM-5-based psychiatric evaluations. The participants were then administered the Visual Reproduction Subtest of the Wechsler Memory Scale, Oktem Verbal Memory Processes Test, Block Design Subtest of the WISC-IV, Judgment of Line Orientation Test and Benton Visual Recognition Test to measure visuospatial skills and working memory functions.

**Results:**

The results showed that 28% of the sample group was diagnosed with inattentive type ADHD (ADHD-IN), while 72% were diagnosed with combined type ADHD. Additionally, 33.33% of participants had CDS+ADHD. When comparing children with CDS + ADHD to those with ADHD alone in terms of visual-spatial and organizational processing abilities, it was found that those with CDS + ADHD were impaired. Children with ADHD-IN also scored lower on a verbal memory test compared to those with combined-type ADHD. On the other hand, anxiety scores were found to be positively correlated with memory functions.

**Conclusions:**

The study found that ADHD subtypes, the presence of CDS symptoms, and anxiety impact the neurocognitive profile of children with ADHD. Further research is needed to understand the various areas of cognitive function that may be affected. The authors declare that they have no conflicts of interest.

**Disclosure of Interest:**

None Declared

